# *Verbesina alternifolia* Tolerance to the Holoparasite *Cuscuta gronovii* and the Impact of Drought

**DOI:** 10.3390/plants2040635

**Published:** 2013-10-18

**Authors:** Bethany Evans, Victoria Borowicz

**Affiliations:** BEES Section, 4120/School of Biological Sciences, Illinois State University, Normal, IL 61790, USA; E-Mail: bevans@iwu.edu

**Keywords:** *Cuscuta gronovii*, focal resource, parasitism, pulse stressed, resource limitation, *Verbesina alternifolia*

## Abstract

Holoparasites are nonphotosynthetic plants that acquire all resources from hosts. The holoparasite *Cuscuta gronovii* is native to much of the US with a broad host range including *Verbesina alternifolia*, an understory perennial. Both species grow in moderate to moist soils and occur in habitats that may experience prolonged or episodic drought. We applied the Wise-Abrahamson Limiting Resource Model (LRM) developed for plant-herbivore relations to examine the effects of pattern of drought stress on tolerance of *V. alternifolia* to parasitism by *C. gronovii*. Individual plants were assigned one of six treatments that were combinations of parasite (none or addition of parasite) and drought stress (well-watered, continuously-stressed, or pulse-stressed). After pulse-stressed plants had experienced two wet-dry cycles all plants were harvested. Parasitism strongly reduced both shoot and root mass and well-watered hosts exhibited the greatest decline, indicating reduced tolerance to parasitism when water was readily available. This is consistent with the LRM if parasitism limits photosynthates available to the host. However, parasitism increased allocation to shoot and this effect did not differ between well-watered and drought-stressed plants, indicating equal tolerance. This outcome is in accord with an alternative prediction of the LRM if hosts are not carbon limited. Total pot productivity was reduced by parasitism and drought stress, and this effect was greater for pulse-stressed than for continuously-stressed hosts. We discuss the applicability of the LRM for understanding the effects of drought on tolerance to parasitism.

## 1. Introduction

Being non-photosynthetic, holoparasites obtain all needed resources from hosts via root-like haustoria [1]. Haustoria bridge the vascular tissues of parasite and host, enabling the parasite to extract water, minerals, and organic compounds produced or acquired by the host at some cost. Parasites can greatly reduce host growth but the impact is likely to be a product of resource supply for the host. Because water is a key resource affecting the ability of a plant to acquire and transport minerals [2], to fix carbon [3], and distribute photosynthates, water availability is likely to affect plant-parasite interactions.

Plants transport assimilates in the phloem by loading solutes at the source, osmotically raising turgor pressure and unloading solutes at the sink, thereby lowering the turgor pressure [4]. In general, stress reduces source strength by reducing photosynthesis, and sink strength by inhibiting growth which diminishes translocation [5]. Consequently, plants experiencing moderate to severe drought stress may show not only slowed nutrient uptake and transport but also a reduction in photosynthate translocation [6] and thus a change in nutrient availability to a consumer.

Although pattern of drought stress is likely to affect plants and the holoparasites attached to them, literature is lacking on parasitic plant-host responses to water stress. Nonetheless, drought-mediated interactions between herbivores and host plants have been examined extensively [7,8,9,10,11,12]. Because herbivores and parasitic plants possess similar feeding preferences [13] and the interactions between parasitic plants and their hosts resemble herbivore-host interactions [14], theory developed for explaining plant-insect interactions may provide insight into holoparasite-plant interactions.

Tolerance describes the ability of a plant to grow and reproduce despite being under attack [15,16,17]. A tolerant plant can sustain damage or injury or support an enemy without showing a significant reduction in growth. While it is often assumed that plants exhibit lower tolerance in stressful environments [16,18], many examples show greater tolerance to herbivory under stressful conditions [19,20,21]. With the goal of providing a general explanation with clear predictions for the relationship between resources and tolerance to herbivory, Wise and Abrahamson [22] proposed the Limiting Resource Model (LRM) of plant tolerance. In our study we use this model as a framework for examining the effects of drought stress on growth of a host supporting a holoparasite.

**Figure 1:**
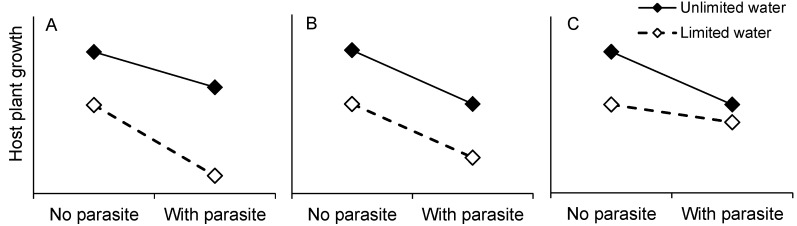
Tolerance slopes describing host plant growth relative to presence of the parasite. (**A**) Greater tolerance to parasite when water is not limiting. (**B**) Equal tolerance to parasite regardless of water stress. There is no interaction of water stress and parasite. (**C**) Reduced tolerance to parasite when water is not limiting.

In this novel application of the LRM, water is the “focal” (manipulated) resource [22]. If parasitism affects the ability of the host plant to acquire enough water, the impact of the parasite should be small when water availability is high and the host plant should express greater tolerance to the parasite when not drought stressed (Figure 1A). Although shoot parasites may attach to xylem and affect water availability, holoparasites can strongly limit host growth by siphoning off photosynthates transported in the phloem [23,24]. We assume carbon is the resource most affected by the holoparasite, *i.e.*, the “alternate” resource in the LRM. If a host plant does not become carbon limited when water availability is high, the parasite’s effect is independent of water and the plant is predicted to be equally tolerant to parasitism at all levels of water (Figure 1B). If carbon becomes the limiting resource when the host plant has abundant water, the parasite’s effect depends upon whether it increases or decreases this carbon limitation for the host. If the parasite increases carbon limitation of the host, then the impact of the parasite is relatively larger when water is readily available than when the host is drought-stressed, leading to lower tolerance to the parasite when water availability is high (Figure 1C). Because they are drawing upon the host’s photosynthates, holoparasites are unlikely to relieve host carbon limitation and so the last outcome outlined in the LRM (greater tolerance at higher focal resource level due to decreased alternate resource limitation) would not apply.

Our experiment was intended to distinguish among three possible responses to parasitism as a function of water stress: greater tolerance, equal tolerance, or reduced tolerance. In addition to observing the effect on tolerance, we also measured effect of drought stress on total productivity (host mass + parasite mass) to determine whether reduced host growth was balanced by increased parasite growth, and whether any depression in overall productivity was altered by the temporal pattern of drought stress.

Our greenhouse experiment focused on *Verbesina alternifolia* (L.) Britton ex Kearney (wingstem) as host and *Cuscuta gronovii* Willd. Ex Schultes (dodder) as parasite. The holoparasite *C. gronovii* is native to much of the US and has a broad host range that includes *V. alternifolia*, an understory perennial. *Cuscuta gronovii* and *V. alternifolia* occur in areas that may experience prolonged or repeated episodes of drought. More severe and extended periods of drought are predicted consequences of an increase in global temperatures [25]. While continuous water stress is characteristic of many experimental studies, pulsed stress treatments may better mimic many natural situations [26,27,28]. Responses of plants to episodic water limitation and underlying mechanisms are not well studied [29]. To our knowledge, how temporal pattern of drought affects plant-holoparasite relations has not been explored. This stands in contrast to studies of plant-herbivore relations, which have focused on herbivore performance (see [27] for review). In particular, phloem-feeding insects are hypothesized to perform better on pulse-stressed plants as a result of increased concentrations of nitrogen that become available after stress is relieved [27]. We conducted a greenhouse experiment with two objectives: (1) to test the pulse-stress hypothesis with dodder as the consumer (data not presented); and (2) to examine how the combined effects of this vascular feeder and temporal pattern of drought affect growth of the host, a native herbaceous perennial. In this paper we present the results of host growth within the framework of the Limiting Resource Model of plant tolerance.

## 2. Results and Discussion

### 2.1. Host Growth

**Figure 2:**
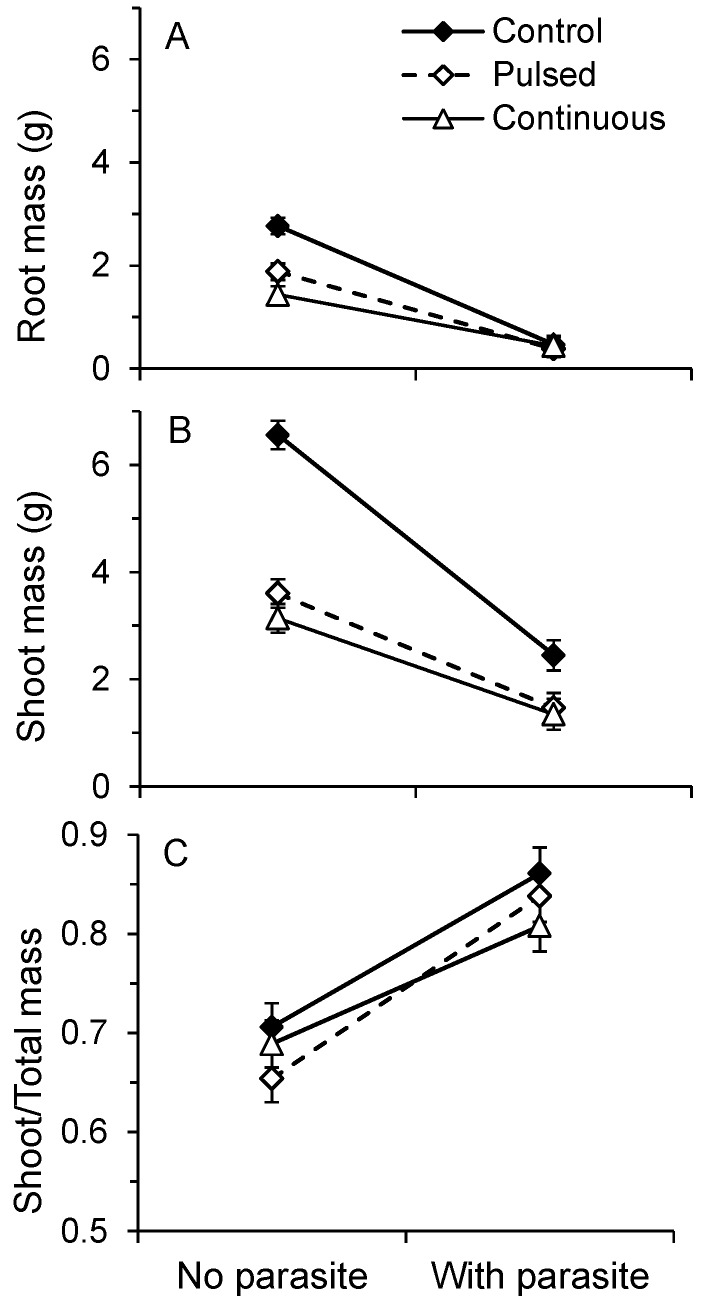
Effect of parasitism on (**A**) root mass, (**B**) shoot mass, and (**C**) the proportion of total mass consisting of shoot in *V. alternifolia* maintained under well-watered (=control), pulse-stressed, or continuously drought-stressed conditions. Values are least squares means (±2 se).

Drought stress and parasitism significantly reduced the growth of the host roots and shoots, but the effect of the parasite differed among drought treatments (Table 1A) and this effect was more strongly expressed in the shoots (larger standardized canonical coefficient). Parasitism more strongly reduced root and shoot growth in control plants (high water availability) than in plants given either the pulse-stressed or continuously-stressed drought treatments (Table 1B, Figure 2A,B). Among plants that experienced drought, parasitism more strongly reduced root mass of pulse-stressed plants (Table 1B, Figure 2A). The impact of parasitism did not differ between pulse-stressed and continuously-stressed plants in terms of shoot mass (Table 1B, Figure 2B).

**Table 1:** (**A**) Multivariate analysis of covariance for effect of drought stress (control (=not stressed), pulsed, continuous), presence or absence of parasite (*Cuscuta gronovii*), and their interaction on total *Verbesina alternifolia* host shoot and root mass using the length of the longest leaf as a pretreatment estimate of host variation, and (**B**) contrasts testing whether the effect of parasitism is consistent across water treatments for host shoot and root dry mass.

**A**	**Pillai’s trace**			**Standardized canonical coefficient**				
	**df**	***F***	***P***	**Shoot**	**Root**	**Shoot 2**		**Root 2**
Leaf length	2,161	18.78	<0.0001	1.844	0.956	-	-	
Water	4,324	42.13	<0.0001	2.935	−0.463	−1.520	2.836	
Parasite	2,161	352.01	<0.0001	1.494	1.281	-	-	
Parasite*Water	4,324	19.84	<0.0001	1.891	0.910	−2.711	2.725	
**B**	***V. alternifolia* shoot mass contrast**			**df**	***F***	***P***		
Control *vs*. Pulsed				1,162	52.29	<0.0001		
Control *vs*. Continuous				1,162	69.78	<0.0001		
Pulsed & Continuous				1,162	1.53	0.2172		
***V. alternifolia* root mass contrast**				**df**	***F***	***P***		
Control *vs*. Pulsed				1,162	23.89	<0.0001		
Control *vs*. Continuous				1,162	61.66	<0.0001		
Pulsed & Continuous				1,162	9.39	0.0026		

The LRM was originally presented as a flowchart [22] and subsequently as a dichotomous key [30] where outcomes depend upon answers to a series of questions. A pivotal question is whether higher focal resource levels cause the alternate resource to become limiting. We assumed the answer is yes, when water is plentiful photosynthesis limits plant growth. This is based on the observations that *V. alternifolia* grow well in full sun, and that light levels in our greenhouse are less than full sun. Given this assumption, the host is predicted to exhibit lower tolerance to the parasite (greater reduction in growth) when water is readily available, as indicated by a statistical interaction. When expressed as dry shoot mass or dry root mass, our results are in accord with this prediction.

Although our results suggest greater tolerance to the parasite when the plants are drought stressed, the interpretation may be less straightforward. We evaluated the raw data because they fit the assumptions of analysis of variance much better than did transformed data. However, biomass is frequently log transformed due to the allometric scaling relationship of plant growth, which is multiplicative rather than additive [31,32]; proportionally similar effects on large *vs*. small plants yield larger absolute differences for the larger plants. The comparatively large reduction in growth of well-watered plants due to parasitism is partly a product of the vigorous growth of plants in this treatment and consequent large absolute effects.

According to optimal partitioning models, plants respond to variation in the environment by partitioning biomass among various organs to capture nutrients, light, water, and carbon dioxide to maximize growth rate [33]. Well-nourished *Cuscuta*-parasitized hosts exhibit reduced root: shoot ratios because the parasite acts as a strong sink relative to the host root [24]. Therefore, demand by parasites for photosynthates would be predicted to shift host allocation to shoot in order to gather more light. Likewise, water limitation would shift allocation to roots in order to gather more water. If photosynthesis does not limit host growth then the LRM predicts no difference in the effect of parasitism at high *vs*. low resource levels, *i.e.*, these effects of parasitism and water availability should be independent. Parasitism increased (*F*_1,162_ = 219.75, *p* < 0.0001) and drought stress decreased allocation to shoot mass (*F*_2,162_ = 5.43, *p = * 0.0052) and the effect of parasitism depended upon the drought treatment (*F*_2,162_ = 3.43, *p = * 0.0348). Although parasitism and water stress were not independent, this interaction arises from the difference between the two stress treatments. Control plants, which were not drought stressed, did not differ significantly from either drought-stress treatment (*p* > 0.05 for each). However, parasitism shifted allocation to shoots more strongly in pulse-stressed plants, which occasionally experienced relief from water limitation, than in continuously-stressed plants (*F*_1,162_ = 6.83, *p* = 0.0098; Figure 2C). Thus, from the perspective of allocation, our results provide some support for two different predictions. The outcome is consistent with the prediction of no effect of drought stress on tolerance to parasitism when the standard is well-watered controls, but consistent with the prediction of reduced tolerance to parasitism with drought stress when drought stress is intermittent.

In sum, our conclusions regarding reduced tolerance *vs*. no change in tolerance to parasitism as a function of water supply remain equivocal. Growing conditions in the greenhouse did not definitively place well-watered hosts in a light environment that limited growth, and growth response of the hosts can be interpreted as meeting either of two predictions of the LRM when water is the focal resource and parasites limit an alternate resource. However, we find no support for a third potential outcome of the LRM, *i.e.*, reduced tolerance to parasitism when plants are drought stressed (Figure 1A), which is predicted if either parasitism affects the host’s ability to acquire water or if parasitism alleviates carbon limitation.

### 2.2. Pot Productivity

**Figure 3:**
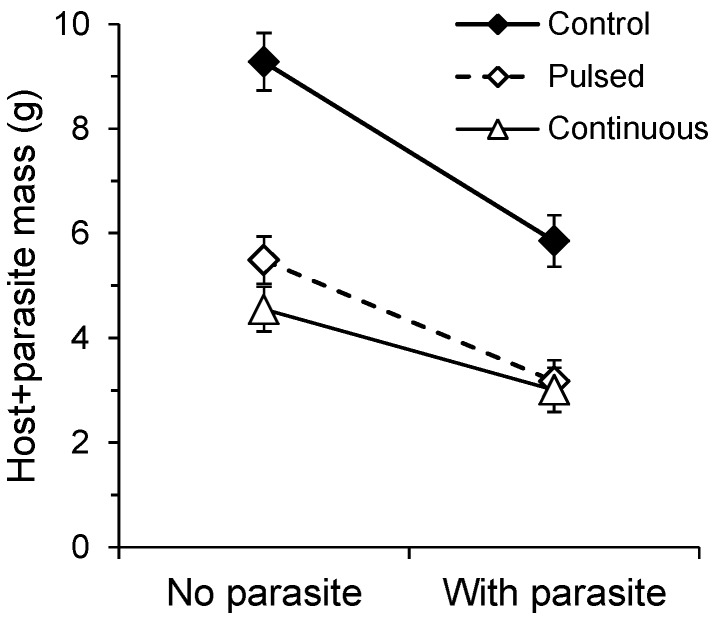
Total productivity from pots of individual *V. alternifolia* with or without dodder and well-watered, pulse-stressed, or continuously drought stressed. Values are back-transformed least squares mean (±2 se) dry mass of host roots + shoots + parasite.

Largest leaf length, a measure of pre-treatment variability among hosts, significantly affected final pot productivity, as measured by parasite + host biomass (*F*_1,162_ = 36.44, *p* < 0.0001). Nonetheless, parasitism strongly reduced total pot biomass (*F*_1,162_ = 162.95, *p* < 0.0001) indicating that the parasite not only shifted resources from the host to itself, but reduced the host’s rate of growth. Drought treatment also reduced total pot biomass (*F*_2,162_ = 146.98, *p* < 0.0001) and altered the effect of parasitism (*F*_2,162_ = 4.24, *p* = 0.0160). The reduction in productivity due to parasitism was significantly greater for well-watered plants than for continuously-stressed plants (*F*_1,162_ = 8.43, *p* < 0.0042; Figure 3) but no other contrasts were significant (*p* > 0.05 for each). These data were log-transformed prior to analysis and so the effect of parasitism was in part a proportional reduction in whole pot productivity.

Parasitism can depress photosynthesis and growth [34], or cause a sink-dependent increase in photosynthesis [35] with reduced productivity when the uptake of photosynthates by the parasite does not balance loss from the host. Parasitism by *Cuscuta* can also produce a total pot yield similar to that from uninfected pots [24]. In our experiment, *C. gronovii* grew especially large on plants well supplied with water [36], and reduced total productivity of these control pots to a greater extent than pots in which the hosts were continuously stressed. Given that a holoparasite imports all photosynthates, robust parasite growth increases sink strength and the demand on the host. Increasing drought stress leads to dehydration avoidance responses, e.g., closure of stomata, decreased rates of transpiration, and depressed photosynthesis, and in turn, reduction of plant growth [37,38]. Compared to plants that were not drought stressed, near shutdown by continuously-stressed plants provided little opportunity for exploitation by the parasite.

### 2.3. Requirements and Utility of the LRM for Holoparasite-Host Interactions

Wise and Abrahamson [22] outline four experimental requirements for testing the LRM. Our study met the first requirement of a full factorial experimental design, where plant performance was evaluated under high and low-resource level and high and low-consumer pressure. A second requirement calls for the focal resource (water) to ultimately limit host fitness. We evaluated tolerance in this experiment by examining host plant biomass as an indicator of fitness. Based on the growth of the host in response to the treatments, we are confident that reduced water availability would indeed have limited fitness [39,40]. A third requirement entails measuring physiological parameters to confirm the resource limited by the consumer. Although we assumed that the parasite limited carbon by obtaining photosynthates via haustorial connections to the host’s phloem, other resources may have been affected as well. Haustorial connections to the host’s xylem could have led to nitrogen or even water limitation by the parasite [6]. However, parasitism increased investment in shoot relative to root which indicates that the parasite limited aboveground processes more than it limited belowground resource acquisition. Research on the congener *C. campestris* has demonstrated that this holoparasite reduces growth of the host both through acquisition of photosynthates and reduction in host photosynthesis [34].

Our experiment did not meet a fourth requirement of the LRM, that consumer-damage levels be consistent across environments. Although hosts were infested with a single parasite, parasites grew significantly better on well-watered hosts, to the extent that parasite growth exceeded host growth [36]. Thus, apparently lower tolerance of parasitism by well-watered plants is, at least in part, an expression of lower resistance to and consequently greater consumption by the parasite. Unlike insect herbivores, which are generally a small fraction of the size of the host, parasitic plants such as *Cuscuta* spp. can greatly exceed the size of an individual host as they grow across multiple hosts. Short of trimming the parasite, this requirement of the LRM would be difficult to meet in many plant-parasite systems. Nonetheless, we believe the LRM provides a useful framework for examining the impact of the abiotic environment on responses of plants to parasites.

## 3. Experimental

### 3.1. Study Organisms

The host species, *Verbesina alternifolia* (Asteraceae) grows from 1–3 m tall in full sun to light shade and moderate to moist soils. This native perennial is frequently found in thickets, woods, and bottomlands throughout the Midwestern and Eastern United States including Illinois [41]. Its common name of wingstem derives from the distinct wing-like structures running the length of the main stem. Known as common or swamp dodder, *Cuscuta* *gronovii* (Cuscutaceae) is the most common and abundant species of *Cuscuta* in North America. Found in moist thickets, roadsides, and fields through the Midwestern and Eastern United States as well as adjacent Canada [42,43], this annual holoparasitehas also been introduced to Europe [44]. The broad host range of *Cuscuta* *gronovii* includes at least 175 native and crop plants [45] and it is a particularly noxious weed in cranberry production, where heavy infestation can reduce yield up to 100% [46].

*Cuscuta* spp. twine around a host and form haustoria that penetrate the host’s vascular tissue [47]. Following attachment, the part of the seedling that existed prior to loop formation withers away and growth is based on resources acquired from the host. Searching hyphae differentiate into either xylem or phloem elements upon contact with the host plant’s vascular bundles [48] through cell recognition at the point of contact [49]. Water and minerals are acquired through connections to both the host xylem and phloem but carbon and nitrogen are obtained mainly through phloem attachments [23,35,50] by means of symplastic transfer [48]. The parasite creates a very efficient sink that deprives the host roots of carbon, leading to increased net photosynthesis of the host and a nitrogen deficit in host shoots and roots [35].

### 3.2. Experimental Procedure

Seeds of the host plant and the parasite were collected in October 2010 adjacent to a prairie near the Mackinaw River in Lexington, IL, USA. *V. alternifolia* seeds were cold stratified for 278 days in a refrigerator in bags containing damp Perlite^TM^. Stratified seeds were subsequently germinated in pairs in small pots. The dodder seeds were cold stratified similarly for 188 days and then placed on the surface of a damp mixture of soil, sand, and Perlite^TM^ to germinate. All seeds were germinated in a greenhouse and were frequently misted and watered to maintain adequate moisture.

*V. alternifolia* seedlings (22 days post germination) were transplanted singly into 180 1.7-L pots filled with 1 L of 1:1 potting medium (MetroMix^TM^ 510:Perlite^TM^). Host plants were randomly assigned one of six treatments that were combinations of parasite (no parasite or addition of a *C. gronovii* seedling 12 days after host transplant) and drought stress (well-watered, continuously drought stressed, or pulse stressed). Details are provided below.

Three pots of each of the six treatments were randomly assigned to 10 tables totaling 18 pots per table. The positions of all pots were rotated on tables and the tables were rotated around the room. Bamboo skewer sticks were placed in all 180 pots. Velcro^TM^ strips were used when necessary to tie aggressively growing parasites to the stick in order to prevent spread to other pots. The greenhouse lights (600 watt high pressure sodium) were on a 14:10 L:D schedule and the temperature range was controlled to remain between 18–24 °C. Eleven and 22 days after transplanting, *V. alternifolia* pots were fertilized with 1 g of Scott’s Rose and Bloom^TM^ (12-4-8 N-P-K), a slow-release granular fertilizer. Total leaf count and length of the longest leaf on each host plant were recorded 13 days after transplant to be used as possible covariates in the analysis.

#### 3.2.1. Parasite Treatment

Six days after ending parasite cold stratification and 13 days prior to initiation of drought treatments, one 3–6 cm *C. gronovii* seedling was haphazardly selected from the tray of seedlings and placed near the host in a shell vial filled with water. These seedlings were monitored twice daily to record date of attachment as well as to replace any *C. gronovii* seedlings that did not survive. Attachment was defined as one entire tight loop around the stem of the *V. alternifolia* host plant.

#### 3.2.2. Drought Stress Treatment

**Figure 4:**
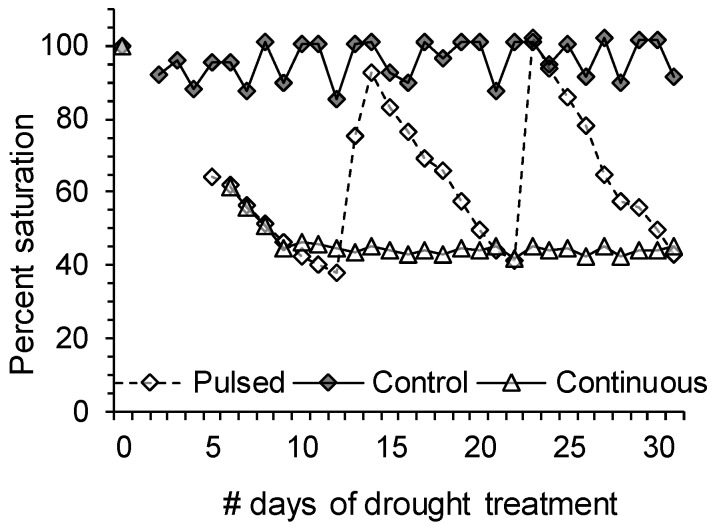
Percent saturation for pots containing single *V. alternifolia* throughout application of water treatments. Values for the two parasite treatments were averaged and the water treatments are expressed as a percent of initial mass of pots at field capacity.

Drought stress treatments began 25 days after host transplant. An initial saturation mass was determined for each pot by flooding the pot with 500 mL of water, waiting 30 min for excess water to drip, and then recording the mass. This saturation mass was used as a benchmark throughout the rest of the experiment. Each of the 120 plants was then randomly assigned one of 6 treatments that were combinations of two factors: drought stress and parasitism. Drought treatments consisted of a control (high water availability), a continuously stressed treatment, and a pulse (intermittently) stressed treatment. Control plants were maintained at >85% of initial saturation (Figure 4). For the control treatment, sentinel pots on each table were weighed daily and all 60 pots in this treatment group received water when one of the sentinel pots fell below 85% initial saturation. Each control was then watered to 100% of mass at initial saturation. Continuously-stressed treatment pots were weighed daily and maintained at 40%–45% of initial saturation. When a pot’s mass fell below 40% saturation it was watered to attain 45% saturation. Pulsed (intermittently stressed) pots received no water until sentinel pots dropped below the lower limit experienced by continuously stressed plants (40% of initial saturation). Severe wilting occurred at 35% of initial saturation and was determined to be the point when recovery was necessary. When one sentinel pot reached 35% initial saturation then all 60 pots received water until they were back to 100% initial saturation. Due to the extended period without water, the pulse-stressed pots were extremely dry and the soil had constricted making watering procedures more difficult. To ensure all water was available for saturation and not lost through the bottom, trays were placed underneath these pots to allow for absorption of runoff. The intermittently-stressed pots were pulsed back to 100% saturation twice, and the experiment was terminated when they were at the point of requiring a 3rd pulse of water. They were not watered at this point in order to keep the pulsed and continuous drought treatments relatively similar to each other in terms of amount of water received throughout the experiment. The water added to each pot in the experiment was recorded daily.

#### 3.2.3. Harvest

The experiment was terminated 57 days after hosts were transplanted, which was 32 days after water stress began. Parasites were carefully detached from host shoots and dried. *Verbesina alternifolia* shoots were cut at ground level, roots were washed to remove all debris from the potting medium, and each part was dried at 60 °C to constant weight.

### 3.3. Statistical Analysis

Because the duration of parasitism could affect final biomass of the host we examined the attachment date of *C. gronovii* prior to analysis and excluded 11 pots in which the parasite took longer than 8 days to tightly loop around the host. Among the pots that were not excluded, over 45% of the attachments occurred within one day and 90% within 3 days. We used days to attachment as a covariate in MANCOVA with water as the main effect to test whether this important event in the host-parasite interaction significantly affected final root and shoot mass of parasitized hosts. The covariate was not significant (*p* = 0.1275) indicating that timing of parasite establishment was not a significant source of variation. The final data set consisted of 26 control, 28 pulse-stressed, and 25 continuously-stressed replicates with parasites, and 30 replicates in each of the drought treatments free of parasites.

To determine the effects of water stress and parasitism on host tolerance, dry root and dry shoot mass of the hosts were analyzed with MANCOVA. Total number of leaves prior to the onset of treatments ranged from 4 to 10 and 90% of plants had 6 or 8 leaves. Preliminary analysis showed that total number of leaves did not explain significant variance of shoot and root biomass (*p* > 0.05). Length of the longest leaf from each host plant did not interact with main effects and explained significant variance in final host biomass measures. Because measures of leaf size are correlated with variation in leaf structure and function [51] we used this variable to account for pre-treatment variation among hosts. MANCOVA was followed by bivariate contrasts to test whether the effects of parasitism varied among water treatments. Generally biomass measurements are log-transformed because treatments that cause proportionately similar responses in large and small plants yield different absolute amounts [28,29]. We analyzed untransformed data because these best met the assumptions of the model. However, analysis of untransformed mass can bias towards finding significant interactions due to the problem of scale [32]. To evaluate this possibility we subsequently analyzed shoot mass divided by total mass and performed contrasts. Finally, to determine whether the parasite reduced overall productivity or merely shifted resources to itself, total host + parasite dry mass was analyzed with ANCOVA using length of the longest host leaf as a covariate to account for pre-treatment variation among hosts. All statistical analyses were performed using SAS 9.2 © 2008 for Windows Version 6.1.7601.

## 4. Conclusions

In a comparison of competing models of plant tolerance to herbivory, Wise and Abrahamson [30] determined that 95% of the studies produced results consistent with predictions of the LRM. When water was the focal resource, plants were more tolerant of herbivory when water was in short supply. Our results with a holoparasite as consumer and water limitation as the focal resource suggest that the LRM is broadly applicable. However, the LRM makes no predictions regarding temporal pattern of stress. While drought-stress treatments imposed in experiments of herbivory are far from standardized, stress is generally applied at a single constant level or a single pulse, with examination of different intensities the exception [28]. Plants may exhibit rapid photosynthetic and growth spurts when watered after drought, but the ability to recover may be affected by previous history of drought or compromised if drought severity impairs biochemical and physiological processes [29]. Some data suggest the severity of drought may be more important to a plant than pulse frequency [26,28] but much depends on how treatments are administered. In our study, overall limitation, rather than temporal pattern of the stress, drove the host response, but pattern of drought affected allocation in response to parasitism.

In addition to affecting individual host growth, parasites significantly reduce crop productivity [52,53] and alter community structure and function [54,55,56]. Consequently, a change in the abiotic environment is likely to alter the magnitude of a parasitic species’ effects at several levels [14]. Climate change models predict more frequent and variable drought conditions [25,57] which will affect plant communities [58,59,60] and parasite-host interactions [60]. Shifts in phenologies and geographic distributions of species in the direction predicted by these models indicate that climate change is indeed occurring [61]. To better understand how climate change will impact holoparasite-host and community relations, future experiments should continue to manipulate parasitism against a background of changing resource availability.
